# Sustainable ionic liquid-assisted cloud point extraction for enrichment of trace copper(II) in water and food samples prior to spectrophotometric determination

**DOI:** 10.1038/s41598-026-56689-x

**Published:** 2026-06-10

**Authors:** Nagwa M. A. El-Bialy, Ayman A. Gouda, El-Sayed I. A. Ghaith, Islam E. Khedr, Ahmed H. Moustafa, Abd-ElNasser A. Mohamed, Ahmed M. Ismaiel

**Affiliations:** 1 Department of Botany and Microbiology, Faculty of Science, Zagazig University, Zagazig, 44519 Egypt; 2 Chemistry Department, Faculty of Science, Zagazig University, Zagazig, 44519 Egypt; 3 School of Specific Education, Zagazig University, Zagazig, 44519 Egypt; 4 Chemistry Department, Faculty of Science, Luxor University, Luxor, Egypt

**Keywords:** Environmentally friendly, Ionic liquid-based cloud point extraction, Copper, Spectrophotometry, Environmental matrices, Greenness, Blueness, Whiteness and carbon footprint profile, Chemistry, Environmental sciences

## Abstract

This work introduces a novel, cost-effective, and sustainable ionic liquid–assisted cloud point extraction (IL-CPE) technique for the enrichment and spectrophotometric quantification of trace copper(II) at 436 nm. The method employs the complexation of Cu(II) with (Z)-4-bromo-2-(((2-hydroxyphenyl)imino)methyl)phenol at pH 6.5, succeeded by extraction with 1-butyl-3-methylimidazolium hexafluorophosphate and Triton X-114. Under optimal circumstances, the approach exhibited remarkable linearity (2.0–300 µg L⁻¹, R^2^ = 0.9997), substantial sensitivity with a preconcentration and enrichment factors 100 and 12.5, respectively, and a minimal detection limit of 0.6 µg L⁻¹. Precision and reliability of the novel IL-CPE method was validated by low relative standard deviation for 100 and 200 µg L⁻¹ (*n* = 10) was 1.6% and 2.0%, respectively, and accuracy was substantiated by the use of certified reference materials and actual environmental samples, encompassing water and food matrices. Moreover, the method’s environmental effect was meticulously assessed utilizing several Green Analytical Chemistry metrics (AGREE, AGREEprep, ComplexMoGAPI, AGSA) and practical indices (BAGI, CACI). The evaluation via the RGB algorithm and Carbon Footprint Reduction Index (CaFRI) verifies that this method is a sustainable, eco-friendly, and very dependable option for regular trace metal detection.

**Supplementary Information:**

The online version contains supplementary material available at 10.1038/s41598-026-56689-x.

## Introduction

The ongoing buildup of metal ions in environmental systems requires effective monitoring measures, particularly when their amounts are not biologically controlled. The main analytical difficulty is identifying trace-level analytes in complicated matrices, necessitating techniques that combine operational simplicity with high efficiency^1,2^.

Copper, particularly Cu(II), is a dual-natured element: it serves as an essential cofactor for metalloenzymes like superoxide dismutase and cytochrome c oxidase, but its dysregulation is linked to neurodegenerative illnesses such as Alzheimer’s and Parkinson’s diseases. Moreover, while Cu(II) is used in medicinal formulations for its antibacterial qualities, excessive exposure may lead to immediate gastrointestinal problems or chronic hepatic and renal failure^3–6^.

A preconcentration procedure is needed for an accurate determination of Cu(II) in environmental samples due to its normally low concentrations^7–10^. Diverse analytical methods have been used for its detection, including FAAS^11,12^, ICP-OES^13^, stripping voltammetry^14^, and spectrophotometric methods^15–18^. The direct measurement of trace copper without preconcentration is often impeded by inadequate sensitivity and matrix interferences. To address these issues, many preconcentration techniques have been developed, including coprecipitation^19^, solid-phase extraction^20–22^, traditional liquid–liquid extraction^23,24^, and membrane filtering^25^ Although successful, several procedures depend on volatile or dangerous organic solvents, which pose considerable environmental and safety issues.

Cloud-point extraction (CPE) has evolved as a more sustainable option to address these restrictions^26–30^. This method utilizes nonionic surfactants that experience phase separation at a certain cloud point temperature, facilitating effective analyte concentration inside a compact surfactant-rich phase. CPE is esteemed for its superior preconcentration ability, economic efficiency, and enhanced operational safety^31–35^. The efficacy of CPE can be significantly enhanced by integrating ionic liquids (ILs) as synergistic extraction agents. Distinguished by their negligible vapor pressure, high thermal stability, and tunable solvation properties, ILs facilitate robust interactions with both metal ions and organic ligands, thereby markedly improving phase separation dynamics and extraction efficiency. Furthermore, their minimal environmental footprint compared to traditional organic solvents reinforces their appeal as sustainable media within the framework of green analytical chemistry^33^.

In this context, spectrophotometry was deliberately chosen for this work owing to its cost-effectiveness, simplicity, and widespread availability in routine laboratories^15–17^. Despite being typically less sensitive than FAAS or ICP-OES, its combination with an effective IL-assisted CPE technique successfully mitigates this disadvantage by offering high enrichment factors, hence allowing precise measurement of trace Cu(II) concentrations^26,27^. This technique is consistent with the tenets of Green Analytical Chemistry (GAC), since it minimizes energy usage and eliminates the need for high-temperature plasma systems or combustible gases linked to FAAS and ICP-OES^31,32,34^. Consequently, the suggested IL-CPE–spectrophotometric technique presents a comprehensive analytical approach that combines environmental sustainability, operational ease, and satisfactory analytical efficacy.

**Fig. 1:**
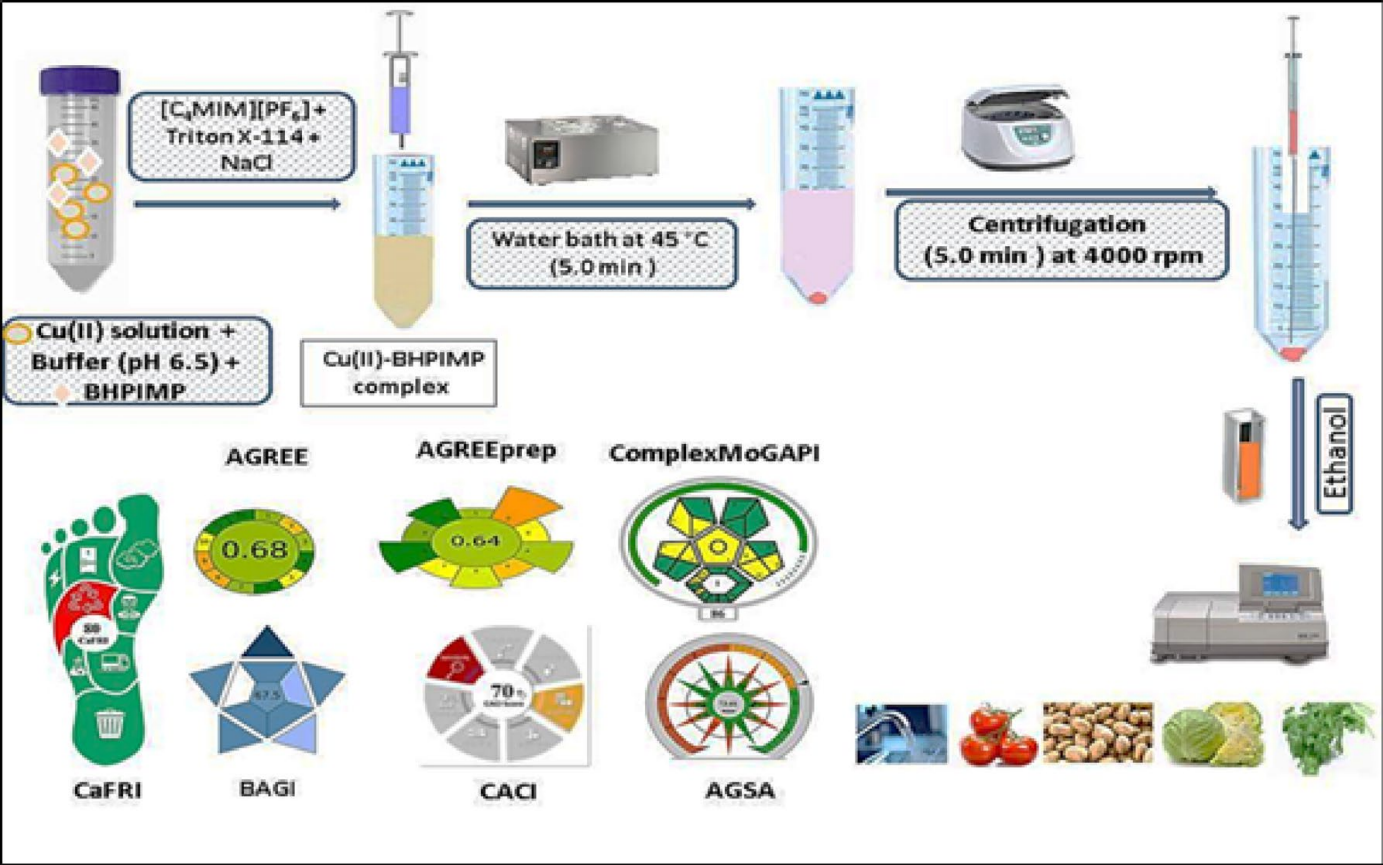
Rationale of the work.

This work presents a novel IL-CPE method using the Schiff base ligand (Z)-4-bromo-2-(((2-hydroxyphenyl)imino)methyl)phenol (BHPIMP) for the preconcentration and quantification of trace Cu(II), in accordance with the 12 principles of Green Analytical Chemistry (GAC). A combined micellar system including [C₄MIM][PF₆] and Triton X-114 was used to attain elevated extraction efficiency (Fig. 1). The procedure was verified using approved reference materials and effectively applied to real environmental samples. The sustainability and overall efficacy of the suggested strategy were thoroughly assessed using many complementing metrics, including AGREE, AGREEprep, ComplexGAPI, and AGSA, in conjunction with BAGI, CACI, RGB12, and CaFRI^36–43^.

## Experimental

### Apparatus

All used apparatus are found in the supplementary materials.

### Materials and reagents

All reagents utilized in this investigation were of high analytical quality. Concentrated nitric acid (65%, v/v) and hydrochloric acid (37%, v/v) from Merck (Darmstadt, Germany) were utilized. A Cu(II) stock solution (1000 µg mL⁻¹) was formulated by dissolving a precisely measured quantity of copper nitrate obtained from Merck (Darmstadt, Germany) in double-distilled water (DDW), supplemented with 1.0 mL of strong nitric acid. Standard working solutions were created daily via sequential dilution of the stock solution with DDW. The IL [C₄MIM][PF₆] was acquired from Acros Organics (Geel, Belgium), while Triton X-114, a non-ionic surfactant, was sourced from Sigma-Aldrich (St. Louis, MO, USA) for evaluation as a viable extraction solvent. A 0.1 M aqueous solution of [C₄MIM][PF₆] was prepared by dissolving the requisite quantity of the IL in 100 mL of DDW with continuous agitation. A 1.0% (v/v) aqueous solution of Triton X-114 was formulated by dissolving 1.0 mL of the surfactant in 100 mL of DDW in a volumetric flask while maintaining steady agitation^44^. Buffer solutions were employed to modify the pH values as previously documented. For the interference research, solutions including diverse ions were prepared from high-purity salts provided by Sigma-Aldrich by suitable dilution with DDW. For interference studies, solutions of various cations were prepared from high-purity salts including chlorides of Na(I), K(I), Ca(II), Mg(II), Fe(III), Hg(II), and Cr(III); nitrates of Na(I), Co(II), Ni(II), Cd(II), Pb(II), and Zn(II); and sulfates of Na(I) and Mn(II). All these reagents were purchased from Sigma-Aldrich (St. Louis, MO, USA) and Merck (Darmstadt, Germany) and were utilized without further purification. Certified reference materials (CRMs), such as TMDA 51.3 and TMDA 53.3 fortified water from the National Water Research Institute, together with spinach leaves (SRM 1570a) from the National Institute of Standards and Technology, were employed to verify the correctness of the proposed analytical approach.

### Synthesis of (z)-4-bromo-2-(((2-hydroxyphenyl)imino)methyl)phenol (BHPIMP)

A 25 mL ethanolic solution of 2-aminophenol (10 mmol, 1.1 g) was subjected to magnetic stirring, subsequently the progressive adding of 10 mL of an ethanolic solution of 5-bromosalicylaldehyde (10 mmol, 2.0 g). The resulting combination was refluxed in a water bath at 70 °C for 6 h. Following the reaction, the mixture was let to setteled to ambient temperature, leading to the emergence of a yellow precipitate. The resultant solid was separated via filtering and refined through recrystallization from ethanol, producing the yellow crystalline substance BHPIMP. The product was ultimately dried in a desiccator containing anhydrous CaCl₂ (Fig. S1).

Yield: 80%; melting point: 196 °C; Anal. Calc. for C_13_H_10_BrNO_2_ (292.13 g mol^− 1^): C, 53.45; H, 3.45; N, 4.79. Found: C, 53.42; H, 3.44; N, 4.76%. FT-IR (KBr), cm^− 1^: ν(C = N) 1625, ν(C = C aromatic) 1587 − 1453, ν(C-O) 1320, and ν(C-Br) 585 (Fig. S2a). ^1^H-NMR (100 MHz, DMSO-d_6_, 25 °C, ppm): σ = 6.88–6.99 (m, 4 H, Ar-H), σ = 7.36 (m, 1H, Ar-H), σ = 7.51 (m, 1H, Ar-H), σ = 7.86 (s, 1H, Ar-N), σ = 7.98 (s, 1H, N = CH), σ = 9.84 (s, 1H, O1H, Exchange with D_2_O), σ = 13.89 (1s, 1H, O2H, Exchange with D_2_O) (Fig. S2b).

A stock solution (1.0 × 10^− 3^ mol L^− 1^) of BHPIMP was created by dissolving a precise quantity of BHPIMP in a minimal volume of ethanol and subsequently completing the volumetric flask to 100 ml with ethanol. The functional solution was obtained by adequate dilution with the same solvent.

### IL-CPE preconcentration procedure

Into a 50 mL centrifuge tube, a 30 mL portion of Cu(II) solution within the concentration interval of 2.0–300 µg L⁻¹ was retained. Afterwards, 5.0 mL of phosphate buffer (pH 6.5) was added to the solution. Then, 2.0 mL of BHPIMP solution (1.0 × 10⁻³ mol L⁻¹), 200 µL of [C₄MIM][PF₆] (0.1 mol L⁻¹), 300 µL of Triton X-114 (1.0%, v/v), and 1.0 mL of NaCl solution (2.0%, w/v) were successively added. The mixture was then filled to the level with DDW and thoroughly mixed. The tubes were subsequently placed in a water bath at 45 °C for 5 min. After incubation, the tubes were transferred to an ice bath and kept for another 5 min, resulting in the formation of a cloudy system. Phase separation was facilitated by centrifugation at 4000 rpm for 5 min. The ionic liquid-rich phase settled at the bottom of the tube, while the aqueous phase was carefully removed using a syringe. Lastly, the remaining ionic liquid–Triton X-114 extract was diluted with ethanol to a total volume of 500 µL and analyzed spectrophotometrically for the Cu(II)–BHPIMP complex at 430 nm.

### Analysis of real sample preparation and CRMs

#### Water samples

Tap, bottled mineral, sea, and waste water were sourced from various Egyptian locations and transferred into clean polyethylene containers. Prior to analysis, samples were passed via 0.45 μm cellulose membrane filters (Millipore Corporation, Bedford, MA, USA) to remove particulate solids. The filtrates were acidified with diluted nitric acid and preserved at 4 °C to maintain sample stability. For mineralization of residual organic constituents, aliquots were treated with 1.0% (v/v) hydrogen peroxide in the existence of concentrated nitric acid (65%, m/m). The optimized IL-CPE protocol was then applied to both collected water matrices and fortified CRMs (TMDA 51.3 and TMDA 53.3). Final Cu(II) concentrations were measured using spectrophotometer.

#### Food samples

Vegetable samples (spinach, tomato, potato, and cabbage) were obtained from local Egyptian markets. Samples were subjected to drying at 80 °C for 1 day and finely ground using an agate mortar. For wet acid digestion, 0.2 g of either SRM 1570a spinach leaves or a prepared food sample was subjected to 2.0 mL of a concentrated HNO₃–H₂O₂ mixture (2:1, v/v) in a beaker. Following a 10-minute period at room temperature on a hot plate the mixture was cooked until a semi-dry residue was achieved. The digest was then dissolved in 10 mL deionized water and a clear solution was obtained by using a 0.45 μm membrane filter. The optimized IL-CPE technique was subsequently implemented, and Cu(II) levels were quantified by spectrophotometer.

To demonstrate the robustness and selectivity of the proposed IL-CPE method, comparative recoveries study using two spiking protocols: before digestion and after digestion was conducted. Before digestion; 100 µg g^−1^ of Cu(II) was added to the solid food samples before the mineralization process. After digestion 100 µg g^−1^ of Cu(II) was added to the acidic sample digest immediately prior to the IL-CPE step.

### Sustainability assessment tools

The ecological efficacy of the method was carefully estimated in accordance with green chemistry principles designed to reduce environmental and health hazards. AGREE, AGREEprep, ComplexMoGAPI, and AGSA. The method’s straightforwardness and practical efficacy were optimized using two tools: BAGI and CACI. The RGB 12 model was employed to assess the multidimensional sustainability profile, including components of whiteness, redness, greenness, and blueness. The CaFRI is a comprehensive greenness valuation instrument that priorities the CaFRI as the primary environmental influence, particularly tailored for the evaluation of validated analytical laboratory methodologies.

## Results and discussion

### The absorption spectra and stoichiometry of the complex

**Fig. 2:**
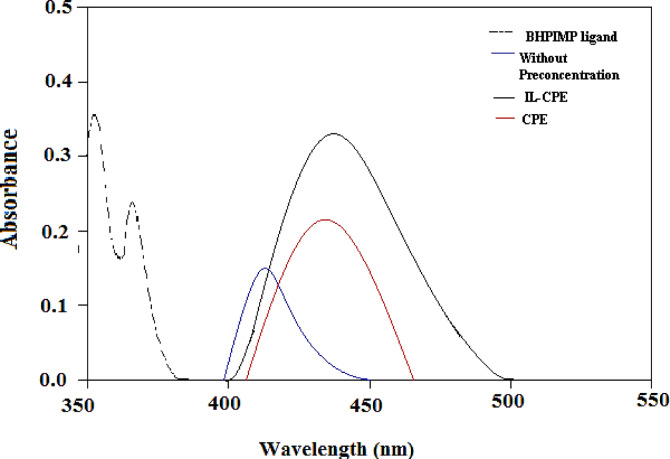
The absorption spectra of the Cu(II)-BHPIMP ligand complex without preconcentration against reagent blank; with CPE and IL-CPE method at optimum conditions with Cu (II) (300 µg L^− 1^).

The absorption spectra of the Cu(II)–BHPIMP complex produced without preconcentration (λmax = 415 nm), after the conventional CPE approach (λmax = 432 nm), and following the IL–CPE method (λmax = 436 nm) are shown in Fig. 2. The observed bathochromic shift in λmax may be explained by changes in the complex’s microenvironment during extraction into the ionic liquid and surfactant-rich phases, which increase the Cu(II)–BHPIMP complex’s stability and hydrophobicity.

The stoichiometry of the complex was investigated using both the mole ratio and the continuous variations (Job’s method) approaches. As shown in Fig. S3a, the maximum absorbance was observed at a mole fraction of 0.5 (C_BHPIMP_ /C_BHPIMP_ + C_Cu(II)_), indicating the formation of a 1:1 (Cu^2+^:BHPIMP) complex. This finding is further supported by the mole ratio plot (Fig. S3b), which shows a clear breakpoint at a ligand-to-metal ratio of 1.0, confirming the 1:1 stoichiometry of the Cu(II)–BHPIMP complex. These findings are consistent with a recently published study using the same ligand under direct spectrophotometric conditions, further validating the proposed complex structure^45^.

### Optimization of the experimental variables

#### Impact of pH

**Fig. 3:**
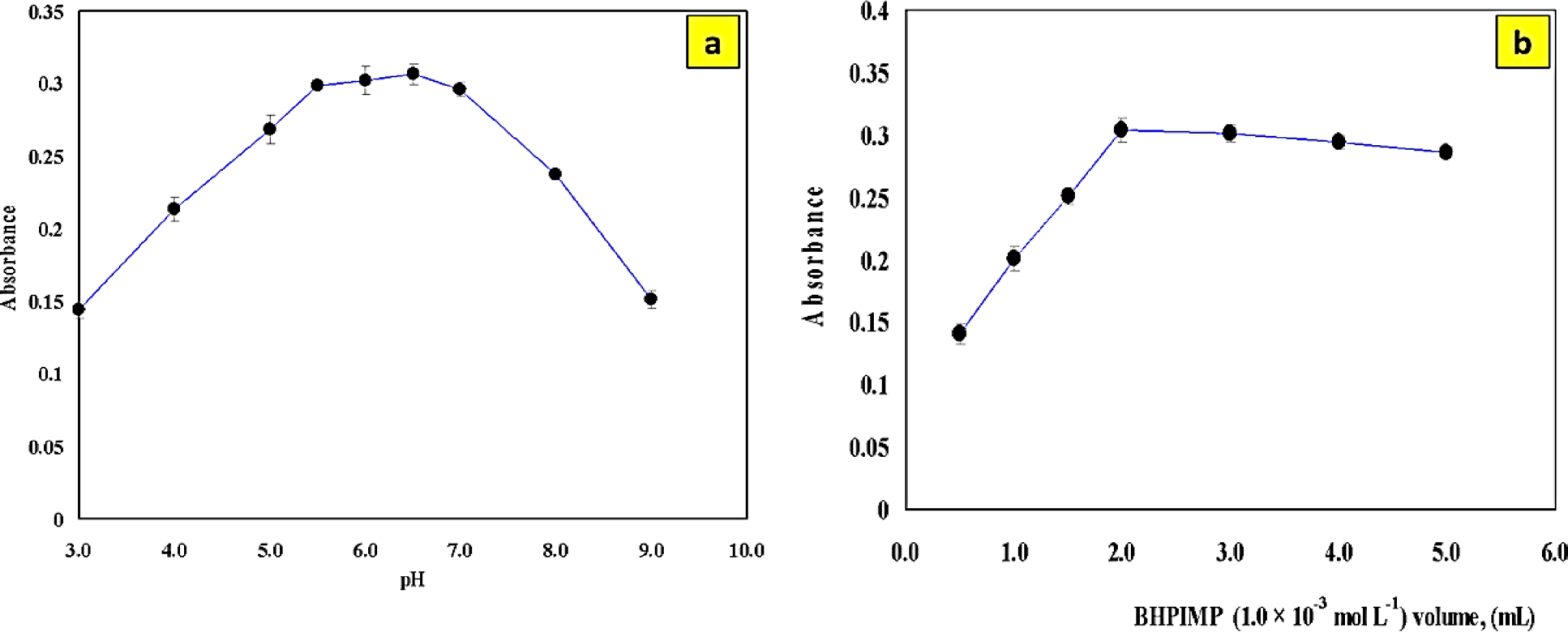
Effect of **a** pH, **b** BHPIMP (1.0 × 10^− 3^ mol/L) volume on the absorbance of Cu(II)-BHPIMP complex through IL-CPE method at Cu(II) concentration (300 µg mL^− 1^).

The solution pH emerged as a decisive parameter governing the stability of the Cu(II)–BHPIMP chelate and the extraction efficiency of the IL-CPE system^28–30^. The extraction performance was therefore systematically evaluated over a pH interval of 3.0–9.0. Figure 3a demonstrate that absorbance intensity steadily escalated with rising pH, reaching a plateau within the range 5.5–7.0, where maximum and reproducible analytical responses were obtained. At inferior pH levels, the reduced signal can be attributed to proton competition with Cu(II) ions for coordination locations on the BHPIMP ligand, thereby hindering complex formation. Conversely, at pH values exceeding 7.0, a decline in absorbance was observed, most likely due to the formation of Cu(OH)_2_ precipitate, which decreases the availability of free Cu(II) for chelation. Based on these findings, 5.0 mL of phosphate buffer solution adjusted to pH 6.5 was selected for all ensuing studies to guarantee optimal complex stability and extraction efficacy.

#### Influence of BHPIMP volume

The concentration of the chelating reagent exerts an essential role in ensuring quantitative complexation of Cu(II). To determine the minimum ligand amount required for complete chelate formation, BHPIMP (1.0 × 10^− 3^ M) levels were varied from 0.5 to 5.0 mL. As presented in Fig. 3b, the analytical response increased steadily with increasing BHPIMP volume up to 2.0 mL, indicating progressive improvement in complex formation. Beyond this volume, no significant enhancement in absorbance or extraction recovery was observed, suggesting that complexation equilibrium had already been achieved. Therefore, 2.0 mL of BHPIMP (1.0 × 10^− 3^ M) was considered sufficient for complete coordination and was adopted in further experiments.

#### Effect of extracting phase composition

**Fig. 4:**
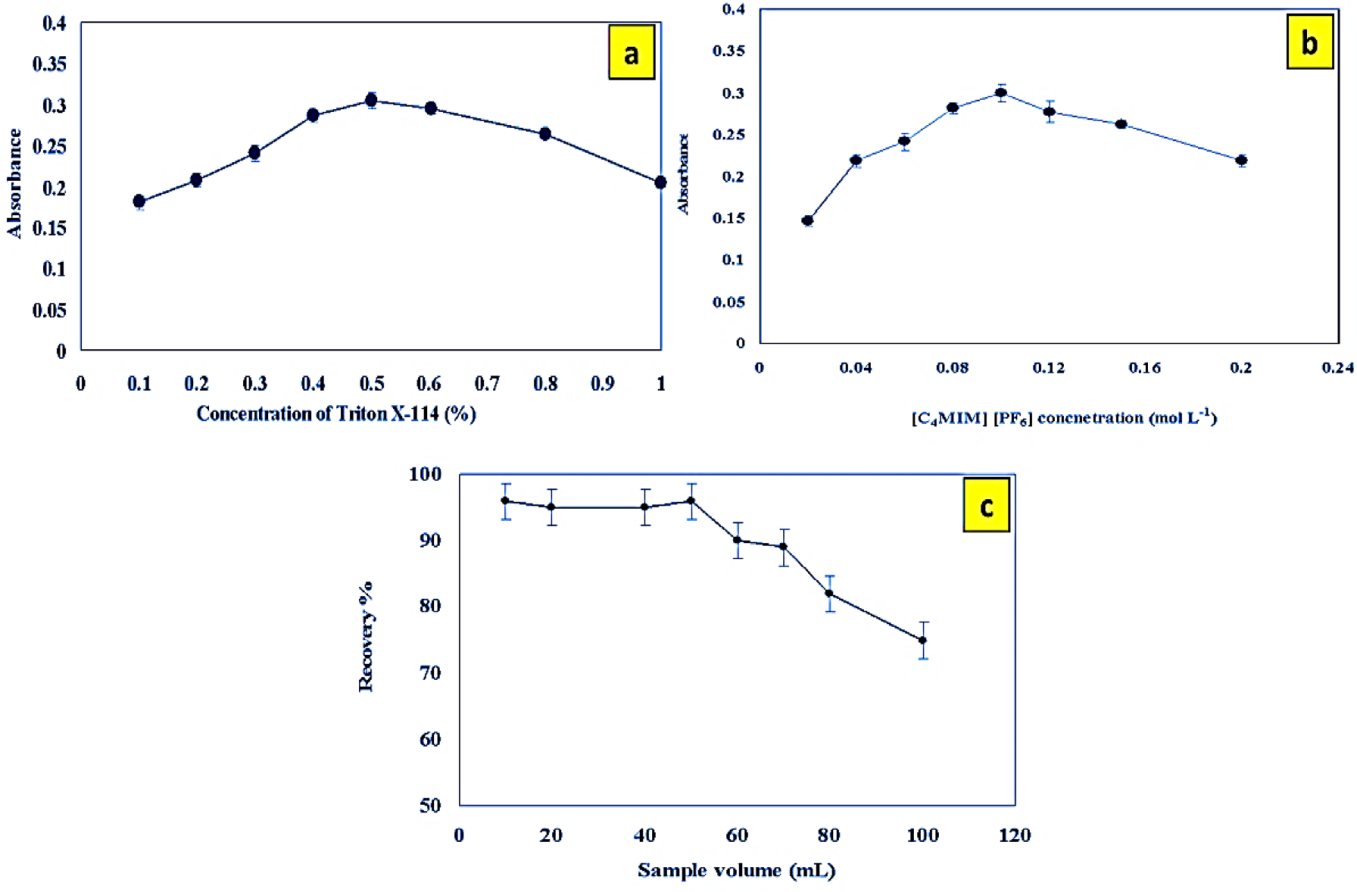
Effect of **a** Triton X-114 concentration, **b** [C_4_MIM][PF_6_] concentration and c sample volume on the absorbance of Cu(II)-BHPIMP complex through IL-CPE method at Cu(II) concentration (300 µg mL^− 1^).

The type and quantity of surfactant significantly influence the extraction proficiency of Cu(II). A number of non-ionic surfactants, namely Triton X-114, Triton-100, and Tween-80, were investigated. The practical findings demonstrated that Triton X-114 exhibited superior performance and provided quantitative recovery of Cu(II). The extraction efficiency was optimized by using Triton X-114 over the range of 0.1–1.0% (v/v). The peak absorbance was obtained at a surfactant concentration of 0.5% (v/v), corresponding to a volume of 300 µL (Fig. 4a). Subsequently, within the range of 0.02–0.2 mol L⁻¹, the effect of the ionic liquid [C₄MIM][PF₆] concentration on the extraction efficacy was examined. The findings demonstrated that optimal extraction efficiency was attained at a concentration of 0.1 mol L⁻¹ with a volume of 200 µL (Fig. 4b). In addition, the incorporation of the ionic liquid was found to improve phase separation and increase both the micellar size and viscosity of the system. Furthermore, all experiments were performed in the occurrence of 1.0 mL of NaCl solution (2.0%, w/v), which was used as a salting-out agent to enhance the extraction process.

PF₄⁻-based ionic liquids like [C₄MIM][PF₄] may raise some stability and environmental issues, despite the fact that ionic liquids are often thought of as more environmentally friendly than traditional organic solvents because of their low vapor pressure and good thermal stability^46^. Imidazolium-based ionic liquids have been linked to toxicity issues, and the PF₄⁻ anion, in particular, may hydrolyze in aqueous or acidic environments, possibly producing dangerous byproducts^47^ However, the amount of [C₄MIM][PF₄] used in this experiment is quite little (200 µL), which greatly reduces these concerns. Additionally, using it improves phase separation and extraction efficiency, which lessens the need for higher amounts of traditional organic solvents. From the standpoint of Green Analytical Chemistry, the method’s overall sustainability is enhanced by lower waste production and solvent use^48^.

#### Effect of sample volume

Analyte volume significantly affects the achievable enrichment factor in microextraction procedures. Model solutions varying from 1.0 to 100.0 mL were evaluated (Fig. 4c). Quantitative recoveries were maintained up to 30 mL; however, volumes exceeding this threshold resulted in incomplete extraction and reduced recovery. Consequently, 30 mL was selected as the maximum applicable volume.

#### Temperature and time influence

Equilibration temperature and incubation time has a significant role on the phase isolation and preconcentration. Therefore, the impact of these two factors on the extraction competence and analytical response was systematically investigated. The equilibration temperature was tested between 30 and 70 °C, while the incubation time was varied between 2.0 and 15 min. The findings showed that the best analytical performance was accomplished at an equilibration temperature of 45 °C with an incubation time of 5.0 min, and these conditions were subsequently adopted for all further measurements.

#### Centrifugation conditions influence

Efficient phase separation following extraction depends strongly on centrifugation parameters. Centrifugation speed was evaluated between 1000 and 5000 rpm. Maximum and reproducible recovery was achieved at 4000 rpm. The influence of centrifugation time was subsequently studied with an interval 2.0–20 min. Complete and stable phase separation was obtained after 5.0 min, with no further improvement observed at longer durations. The best centrifugation condition for all following trials was determined to be 4000 rpm for 5.0 min.

#### Effects of diluent

Before spectrophotometric measurement, the surfactant-rich phase elevated viscosity must be reduced using an appropriate diluting solvent. Therefore, the influence of several diluents, including methanol, ethanol, acetone, tetrahydrofuran (THF), and acetonitrile, was investigated within a volume range of 0.5–3.0 mL. The findings revealed that ethanol provided the most suitable dilution effect, with an optimum volume of 500 µL. Under these conditions, the preconcentration factor, known as the ratio between the initial sample volume and the final volume of the surfactant-rich phase, was calculated to be 60 using the proposed method.

#### Complexation and IL-surfactant synergism

The elevated extraction efficiency and selectivity of the proposed technique are dictated by a twofold mechanism that encompasses the coordination chemistry of the BHPIMP ligand and the synergistic interactions within the IL–surfactant mixed micellar system.

**Fig. 5:**
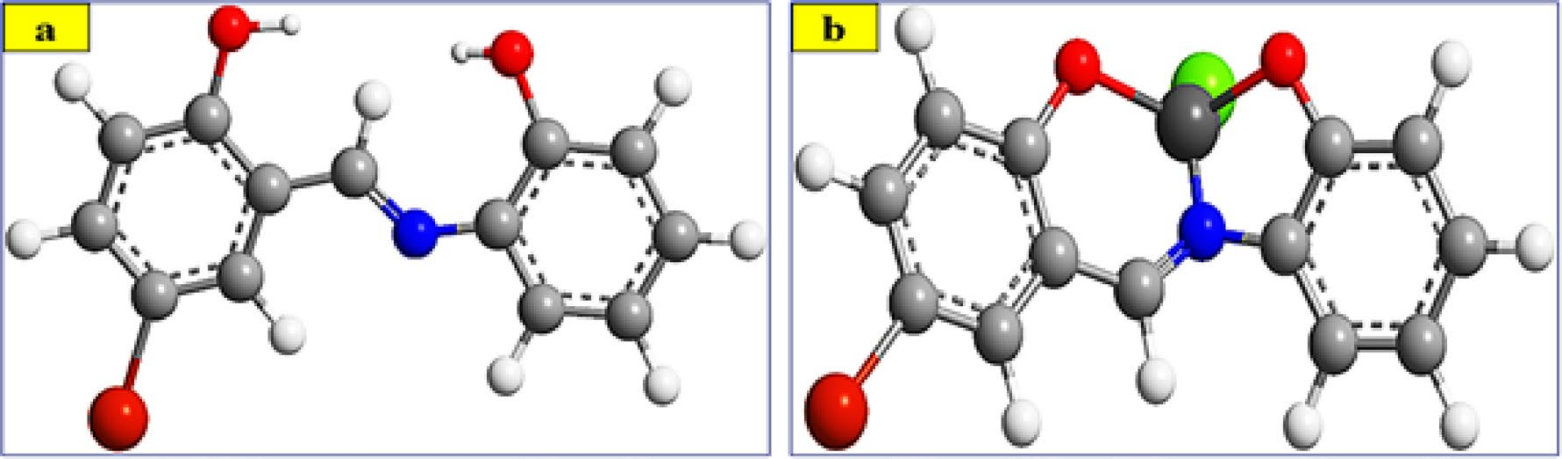
**a** Structure of BHPIMP ligand and **b** Cu-BHPIMP complex.

The selectivity for Cu^2+^ is chiefly due to the structural properties of the Schiff base ligand, BHPIMP, the complexation entails the chelation of Cu^2+^ through the azomethine nitrogen atom (C = N) and the phenolic oxygen, resulting in a stable chelate ring structure with square planar geometry that provides considerable thermodynamic stability and facilitates effective sequestration from the aqueous environment as corroborated by literature on analogous scaffolds^45^. Figure 5 indicates the structure BHPIMP ligand and its Cu-BHPIMP complex.

**Fig. 6:**
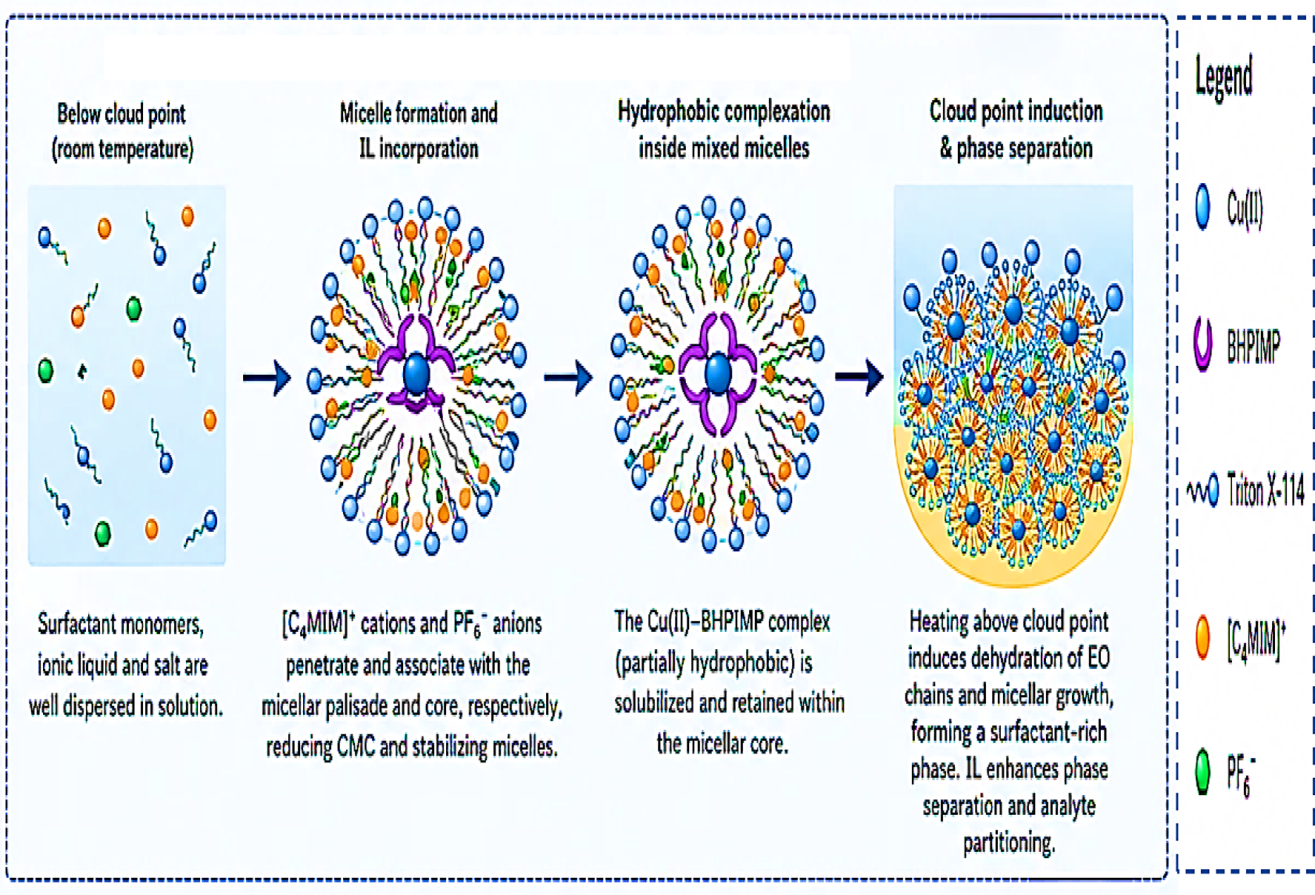
Proposed mechanism of [C_4_MIM][PF_6_]-Triton X-114 CPE of Cu(II) with BHPIMP.

Upon the formation of the Cu-BHPIMP complex, its quantitative recovery is facilitated by the synergistic interaction in which the ionic liquid (IL) [C_4_MIM][PF_6_] functions as a micellar modifier inside the Triton X-114 system. The imidazolium cations (C_4_MIM^+^) intercalate within the palisade layer of non-ionic surfactant micelles, significantly diminishing steric hindrance and electrostatic repulsion among the polyoxyethylene (POE) headgroups, thereby decreasing the critical micelle concentration (CMC) and establishing a more resilient hydrophobic environment. Moreover, the presence of IL aromatic rings enhances π-π stacking interactions with the phenyl rings of the BHPIMP ligand, substantially augmenting the solubilization ability of the micellar core, Fig. 6. The IL-mediated process, along with the dehydration of POE chains during heating, enhances cloud-point phase separation, resulting in a denser surfactant-rich phase that optimizes the preconcentration factor and maintains the thermodynamic stability of the entrapped complex^49–52^.

### Selectivity studies

The enhanced affinity for Cu^2+^ compared to other divalent transition metals (such as Ni^2+^ and Zn^2+^ aligns with the Irving-Williams series^53^, which suggests that d^9^ ions form more stable complexes with nitrogen and oxygen donor ligands. The inherent stability is enhanced by the Jahn-Teller effect, which causes a tetragonal distortion in the copper complex, resulting in a more favorable coordination square planar geometry as shown by DFT simulations in the reference study^45^.

The practical selectivity was rigorously assessed by analyzing the impact of frequently encountered matrix components on Cu^2+^ recovery. Numerous possibly interfering ions were added under ideal circumstances to an aliquot containing 300 µg mL^− 1^ Cu(II), and the results are described in Table 1. The tolerance limit was established as the highest concentration of an extraneous ion that produces a relative inaccuracy within ± 5%. According to these criteria, the suggested approach exhibited exceptional resilience, achieving quantitative recovery of Cu^2+^ (surpassing 95%) in the presence of substantial amounts of competing ions. No statistically significant interference was detected, confirming the method’s enhanced selectivity and suitability for quantifying Cu^2+^ in intricate multicomponent environmental and food matrices.

**Table 1:** Effect of foreign ions on the recoveries of Cu(II) ions utilizing the developed IL-CPE approach (*N* = 3.0).

Interfering ions	Added as	Tolerance limit (mg L^− 1^)	Recovery (%) ^a^
Na^+^	NaCl	3000	95.5 ± 2
K^+^	KCl	3000	97.0 ± 3
Ca^2+^	CaCl_2_	3000	98.0 ± 1
Mg^2+^	MgCl_2_	1000	97.0 ± 3
Cl^−^	KCl	2000	99.0 ± 2
SO_4_^2−^	Na_2_SO_4_	2000	96.5 ± 1
NO_3_^−^	NaNO_3_	3000	95.0 ± 1
Fe^3+^	FeCl_3_	100	96.5 ± 3
Al^3+^	Al(NO_3_)	100	95.5 ± 4
Mn^2+^	MnSO_4_. H_2_O	20	98.0 ± 2
Hg^2+^	HgCl_2_	20	96.0 ± 1
Cr^3+^	CrCl_3_.6H_2_O	50	97.5 ± 2
Co^2+^	Co(NO_3_)_2_	10	98.0 ± 2
Ni^2+^	Ni(NO_3_)_2_.6H_2_O	10	95.0 ± 4
Cd^2+^	Cd(NO_3_)_2_.6H_2_O	10	96.0 ± 2
Pb^2+^	Pb(NO_3_)_2_	10	97.0 ± 3
Zn^2+^	Zn(NO_3_)_2_	10	95.5 ± 1

^a^Mean ± standard deviations. influence of BHPIMP.

### Analytical performance

Excellent linearity was attained across the concentration range of 2.0–300 µg L⁻¹ with the optimized extraction conditions mentioned above. With a correlation value of R^2^ = 0.9997, the calibration equation was written as A = 1.2 × 10^-3^C – 8.0 × 10^− 4^, where A stands for absorbance and C for Cu(II) concentration (µg L⁻¹). The relationships 3σ/k and 10σ/k, where k is the slope of the calibration graph and σ is the standard deviation of 10 blank data, were used to calculate the detection limit (DL) and quantification limit (QL)^54^. It was found that the DL and QL were 0.60 and 2.0 µg L⁻¹, respectively. The low detection limit validates the existing IL-CPE protocol’s appropriateness for trace-level Cu(II) measurement in actual matrices and shows how sensitive it is. Two metrics were used to further analyze enrichment performance: the consumptive index (CI) and the enrichment factor (EF). The EF was determined to be 20, which is defined as the ratio of the slopes of calibration curves produced with and without the preconcentration phase. The CI was 1.5, which was calculated using the formula CI = Vs/EF (where Vs is the sample volume). Replicate studies (*n* = 10) at doses of 100 and 200 µg L⁻¹ were used to assess method precision. Excellent repeatability and methodological stability were demonstrated by the corresponding relative standard deviation (RSD%) values of 1.60% and 2.0%, respectively, with a recovery percentage ranging from 97.0 to 100%.

Also, the molar absorptivity coefficient (ε_max_) for this study was calculated based on the calibration curve. The ε_max_ value was found to be ε_436_ = 7.474 × 10^4^L mol^− 1^.cm^− 1^ based on the slope calibration curve, which plots absorbance vs. molar concentration.

To further highlight the advantages of the developed IL-CPE method, a comparative study was conducted between the proposed IL-CPE method with CPE method and direct spectrophotometric method without preconcentration and the analytical characteristics were listed in Table 2.

**Table 2:** Comparison of the analytical characteristics between the proposed IL-CPE method and the CPE method and direct spectrophotometric method (without preconcentration) for Cu(II) determination.

Parameters	Without preconcentration	CPE	IL-CPE
linearity range (µg L⁻¹)	100–2000	10–600	2.0–300
Calibration equation^a^			
Slope	0.00006	0.0003	0.0012
Intercept	0.0008	0.0146	− 0.0008
Correlation coefficient (*r*)	0.9962	0.9985	0.9997
Molar absorptivity (L mol^− 1^ cm^− 1^)	4.026 × 10^3^	1.57 × 10^4^	7.474 × 10^4^
LOD (µg L⁻¹)	30	3.0	0.60
LOQ (µg L⁻¹)	100	10	2.0
Reproducibility (RSD %) (*n* = 6)	(500 µg L⁻¹) 3.4 (1000 µg L⁻¹) 4.5	(40 µg L⁻¹) 3.0 (80 µg L⁻¹) 3.80	(100 µg L⁻¹) 1.60 (200 µg L⁻¹) 2.0
Recovery%	95.8–98.0	96.0–98.0	97–100
Preconcentration factor (PF)	–	30	60
Enrichment factor (EF)	–	6.0	20
Consumptive index (CI)	–	5.0	1.5

^a^A = a+ bC, where C is the concentration of Cu(II) in µg L^− 1^.

### Method accuracy and validation

Method accuracy and reliability were validated through the analysis of certified reference materials (CRMs) representing diverse and complex matrices: TMDA-51.3 and TMDA-53.3 fortified water and NIST SRM 1570a (Spinach Leaves), which serves as a representative complex biological matrix. To address potential matrix effects, the Standard Addition Method was employed at three concentration levels. We specifically clarify that while this approach was utilized to mitigate non-additive matrix interferences, the high selectivity of the developed methodology against additive systematic errors (such as spectral overlaps) is attributed to the inherent specificity of the BHPIMP ligand. Interference studies confirmed that common co-existing ions in these matrices do not form absorbing complexes at λmax = 436 nm under the optimized pH (Table 1), thus preventing the superposition of absorption spectra that the standard addition method alone cannot eliminate.

The obtained recoveries were in good agreement with the certified values, ranging from 96.40% to 97.54% (Table 3), confirming the reliability of the proposed procedure. The observed accuracy reflects the combined effect of the optimized IL-CPE conditions and the inherent affinity of the BHPIMP ligand toward Cu(II), which contributes to improved analytical selectivity.

**Table 3:** Validation results for Cu(II) determination in CRMs (*N* = 3.0).

CRMs	Certified value (µg L^− 1^)	Found^a^ (µg L^− 1^)	RSD (%)	Recovery(%)
TMDA-53.3 fortified water	308	300 ± 3.60	1.20	97.40
TMDA-51.3 fortified water	89.2	86.0 ± 2.0	2.33	96.40
NIST 1570a Spinach Leaves	12.2 ± 0.6	11.90 ± 0.20	1.68	97.54

^a^Mean ± standard deviation.

### Applications

The practical implications of the suggested approach were further illustrated via the investigation of various water samples and acid-digested food samples. Known amounts of Cu(II) were spiked into the samples to evaluate accuracy via the standard addition approach. Table 4 summarizes that recovery values varied from 95.0% to 100.90%, with RSD values below 4.0%. These results validate that the approach provides reliable quantification, excellent reproducibility, and effective enrichment of trace Cu(II) in environmental matrices.

**Table 4:** The results for the preconcentration of Cu(II) in real samples using the proposed IL-CPE approach (*N* = 3.0).

Water sample	Added (µg L^− 1^)	Found^a^ ± SD (µg L^− 1^)	Recovery (%)^b^	Food sample	Added (µg g^− 1^)	Found^a^ ± SD (µg g^− 1^)	Recovery (%)^b^
Mineral	0	< DL^c^	–	Spinach	0	12.0 ± 0.25	–
	100	96.0 ± 1.10	96.0		100	106.40 ± 1.40	95.0
	200	194.0 ± 2.30	97.0		200	206.0 ± 2.90	97.20
Tape	0	4.0 ± 0.1	–	Tomato	0	9.0 ± 0.30	–
	100	98.80 ± 1.50	95.0		100	106.0 ± 1.0	97.25
	200	200.0 ± 3.60	98.0		200	201.0 ± 2.60	96.20
Well	0	< DL^c^	–	Potato	0	4.0 ± 0.10	–
	100	96.0 ± 1.90	96.0		100	101.20 ± 0.50	97.30
	200	298.0 ± 3.40	99.0		200	202.0 ± 2.20	99.0
Waste	0	15.0 ± 0.22	–	Cabbage	0	4.0 ± 0.14	–
	100	112.0 ± 0.80	97.40		100	102.0 ± 0.90	98.0
	200	217.0 ± 2.90	100.90		200	196.0 ± 1.70	96.0
Sea	0	28.0 ± 0.31	–	Coriander	0	5.0 ± 0.11	
	100	123.50 ± 0.74	96.50		100	102.40 ± 0.75	97.50
	200	225.0 ± 2.40	98.70		200	202.0 ± 2.30	98.50

^a^Mean ± standard deviation. ^b^Recovery% = [Observed value of Cu(II) / Expected value of Cu(II)] × 100. ^c^DL: Detection limit.

The robustness and selectivity of the proposed IL-CPE method was studied and the recoveries were ranged from 95.0 to 98.0% (before digestion) and from 95.5 to 99.0% (after digestion). The close agreement between these values confirms that the digestion procedure effectively mineralizes the organic matrix and The IL-CPE step acts as a highly efficient clean-up tool, ensuring that Cu(II) is selectively preconcentrated into the surfactant-rich phase without spectral overlap from residual organic components.

### Multidimensional sustainability assessment

The sustainability of the suggested IL-CPE technology was assessed using a multi-metric approach. Rather of depending on a solitary indication, eight separate metric tools-AGREE, AGREEprep, ComplexMoGAPI, AGSA, BAGI, CACI, RGB, and CaFRI-were amalgamated to provide comprehensive validation. This thorough evaluation seeks to eradicate the bias present in singular metrics and provides a sophisticated comprehension of the compromises between analytical precision and ecological accountability.

#### Environmental greenness and carbon efficiency

**Fig. 7:**
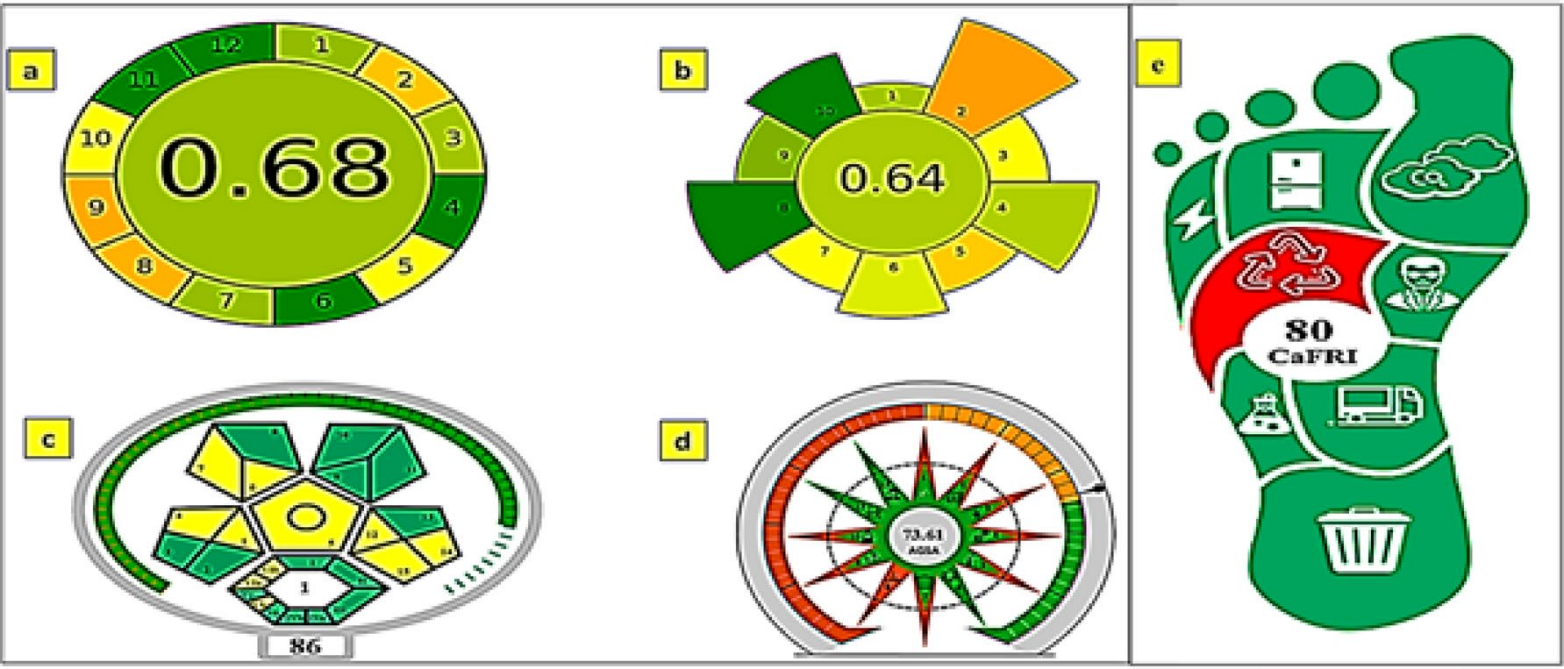
**a** AGREE, **b** AGREEprep, **c** ComplexMoGAPI, and **d** AGSA pictographs, and e Carbon Footprint Reduction Index (CaFRI).

Four complementing metrics were used to validate the Greenness of the methodology. The AGREE metric^36^(https://mostwiedzy.pl/AGREE) yielded a basic score of 0.68 (Fig. 7a), while a compiled version of the open access software of AGREEprep can be obtained from mostwiedzy.pl/AGREEprep^37^. AGREEprep with a score of 0.64 (Fig. 7b), affirmed that the sample preparation, the most crucial phase is optimized for low solvent use. To guarantee that no toxicological effects were neglected, ComplexMoGAPI software is available as an open source on bit.ly/ComplexMoGAPI^38^ (Score: 86, Fig. 7c) and AGSA is available as an open source at bit.ly/AGSA2025^39^ (Score: 73.61, Fig. 7d) were used, indicating that the predominant environmental impact arises from instrumental energy rather than chemical waste. Furthermore, the CaFRI index (available at bit.ly/CaFRI)^43^ (score: 80, Fig. 7e) unequivocally validated the method’s negligible carbon footprint, underscoring its adherence to modern climate-conscious laboratory standards. The reported CFP value pertains solely to the direct energy consumption of laboratory activities (heating, centrifugation, and spectrophotometric measurements) and omits upstream effects like reagent production and transportation; thus, it signifies an operational footprint rather than a comprehensive cradle-to-gate life cycle assessment^36–39^. The method was evaluated comprehensively and quantitatively using the Carbon Footprint of Analytical Methods for Relative Environmental Impact (CaFRI) tool^43^, as illustrated in Fig. 7e. The CaFRI assessment validated the superior environmental profile, emphasizing benefits such as reduced energy consumption with analysis duration of fewer than 10 min, a minimal carbon footprint from instrument power usage, the elimination of nitrogen gas, negligible waste production, and the implementation of a semiautomatic system necessitating only a single operator. These complementing evaluations bolster the environmental sustainability of the devised approach and its appropriateness for application in eco-friendly quality control laboratories.

These findings demonstrate a favorable to relatively high degree of environmental sustainability in comparison to traditional analytical procedures. The suggested method is not completely ecologically friendly since it involves the use of ethanol, nitric acid, digesting reagents, and entails significant energy use. While acid digestion utilizing HNO₃/H₂O₂ guarantees effective matrix decomposition, it marginally diminishes the method’s overall sustainability; thus, more environmentally friendly alternatives like microwave-assisted digestion, enzymatic treatment, or ultrasound-assisted extraction may be explored in future research to improve sustainability.

The synthesis of the BHPIMP ligand was assessed using green chemistry criteria to guarantee a comprehensive sustainable approach. The method exhibited a remarkable Atom Economy of 94.19%, signifying that almost all reactant atoms are efficiently integrated into the end structure, with water being the only stoichiometric waste^55^. The computed E-factor^56^ was 12.12, encompassing the mass of all chemicals. This number is comfortably situated within the acceptable range (5–50) for fine chemical synthesis, indicating that the ligand production adheres to green analytical principles by reducing environmental impact and using sustainable solvents.

#### Applicability assessment

**Fig. 8:**
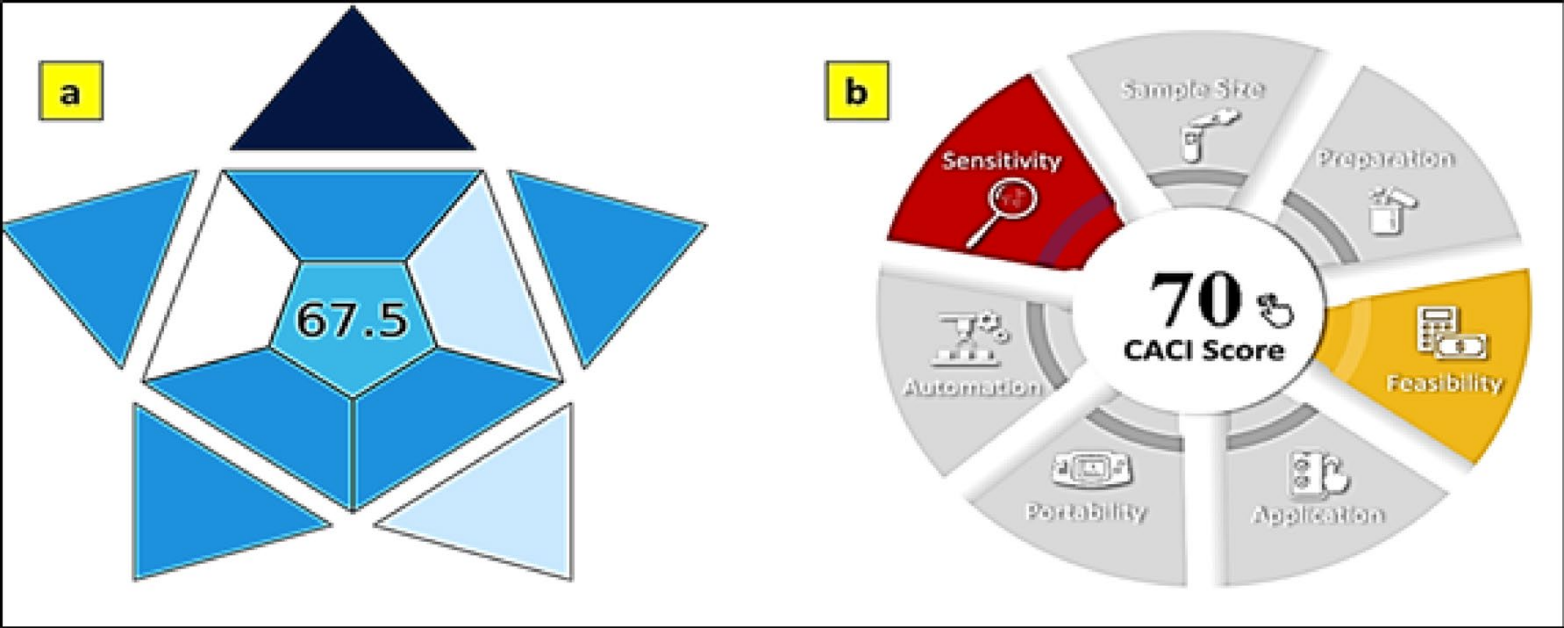
Applicability assessment tools **a** BAGI pictograph and **b** CACI framework.

The method’s functionality for everyday usage was assessed by the evaluation of its Blueness. The BAGI index (score: 67.5, Fig. 8a) indicates adequate throughput. BAGI open-source application was created (mostwiedzy.pl/bagi)^40^. To address elements like as automation and portability not included by BAGI, the CACI framework was used, resulting in a score of 70 (Fig. 8b). The software is available as an open source at bit.ly /CACI2025 ^41^. This dual evaluation verifies that the IL-CPE technique maintains a balance between environmental sustainability and practical applicability in real-world enviro nmental monitoring^40,41^.

#### Whiteness evaluation (RGB Algorithm)

**Fig. 9:**
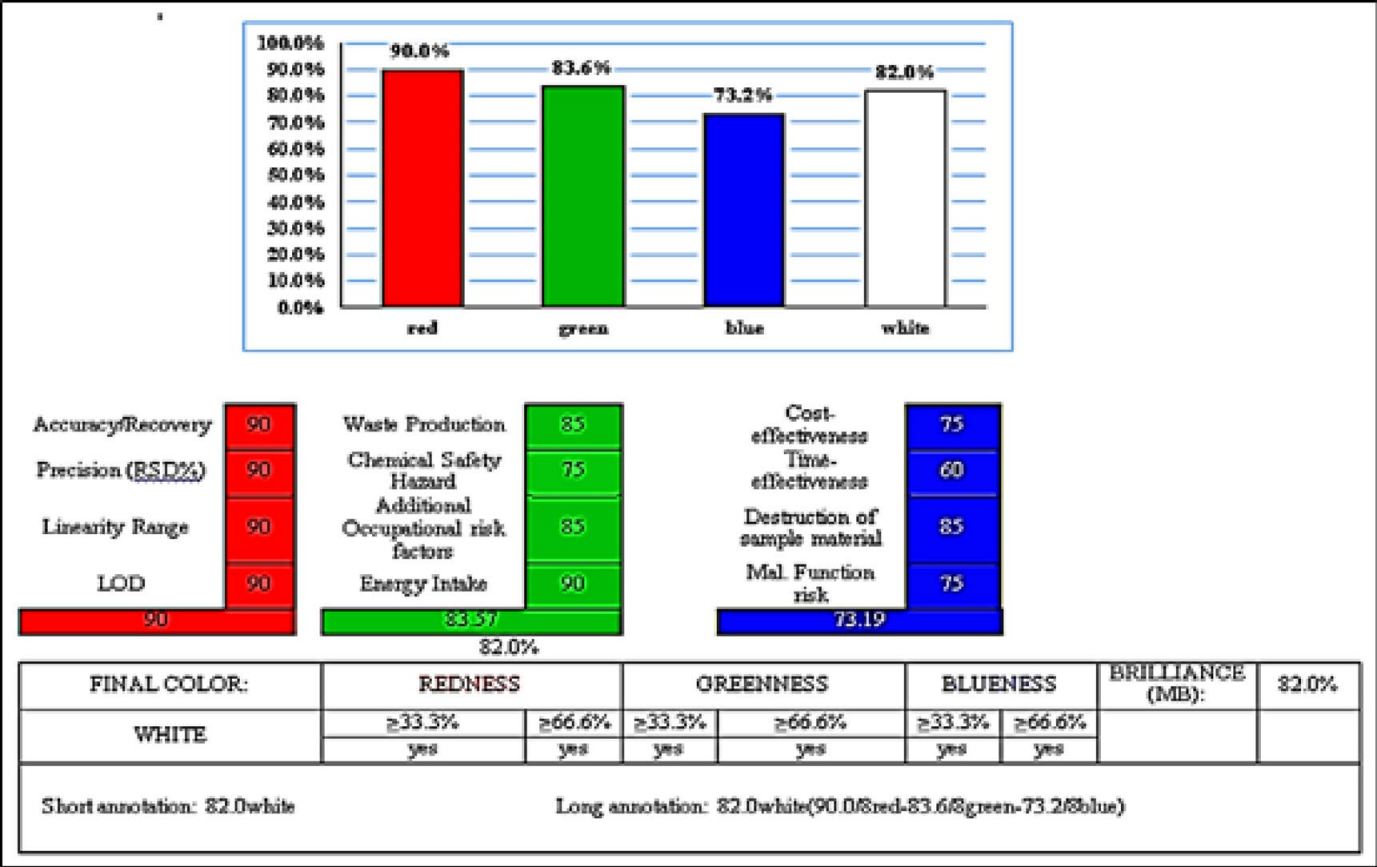
Degree of whiteness via RGB 12 assessment tool.

The RGB method was used to analyze the synergistic and competitive interactions among the three pillars of White Analytical Chemistry (WAC), transcending simple numerical scoring. The approach exhibits an overall Whiteness score of 82.0% (Fig. 9), indicating a strategic equilibrium of analytical performance (Red), environmental impact (Green), and practicality (Blue). Maximizing sensitivity (DL: 0.60 µg L⁻¹) and accuracy (RSD < 3%) often requires complicated or toxic chemicals; however, the use of the IL-CPE method allowed enhanced performance without a corresponding increase in environmental impact. A calculated trade-off is evident, wherein the selection of [C_4_MIM][PF_6_] and Triton X-114 despite their known environmental persistence and hydrolytic considerations was prioritized due to their superior extraction kinetics and ideal cloud point (23–25 °C), which ensure high efficiency without the excessive energy consumption required by more benign alternatives. This minor decrease in Greenness scores was deemed acceptable to substantially enhance Red performance (DL: 0.60 µg L⁻¹; RSD < 3%); this is validated as a beneficial compromise, as the improvements in detection limits and enrichment factors considerably surpass the negligible chemical footprint resulting from the use of microliter volumes. Concurrently, the Blue dimension guarantees that these analytical and ecological innovations do not compromise operational efficiency or economic feasibility, preserving the productivity essential for routine analysis. The RGB 12 is also based on a freely available Excel spreadsheet^42^.

#### Integrated sustainability interpretation

**Fig. 10:**
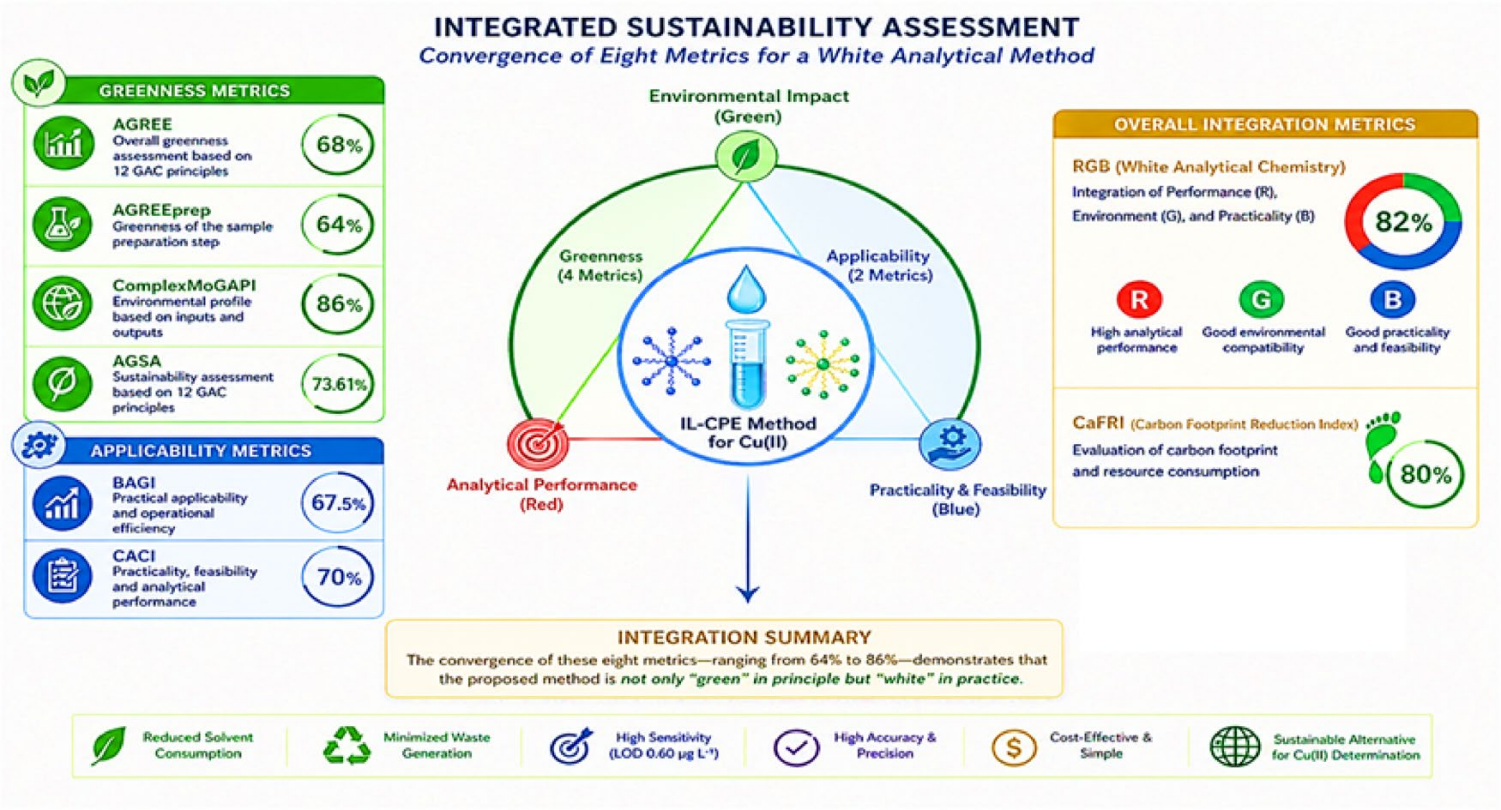
Integrated sustainability assessment of the proposed IL-CPE method using multi-metric evaluation.

The used sustainability evaluation methodologies were analyzed within a unified framework, as shown in Fig. 10. AGREE-based metrics delineate the greenness profile, while ComplexMoGAPI and AGSA examine environmental impact and resource efficiency. BAGI and CACI evaluate practical application (blueness), whereas RGB and CaFRI provide comprehensive insights into overall sustainability and carbon footprint. The alignment of these complimentary indicators, with scores between 64% and 86%, underscores a consistent and well-rounded sustainability profile. This agreement across several instruments serves as a cross-validation process, verifying that the suggested IL-CPE approach is both environmentally sustainable in theory and effective in reality. Thus, the technique constitutes a dependable, sensitive, and sustainable analytical approach for the determination of trace Cu(II), successfully reconciling environmental impact, analytical efficacy, and practical application.

### Comparative assessment

The choice of BHPIMP as the complexing reagent is strategically warranted due to its synthetic simplicity, structural design, analytical efficacy, and sustainability, especially when contrasted with the ligands shown in Table 5.

BHPIMP is synthesized using a direct one-step Schiff base condensation in ethanol at 70 °C, followed by filtering and recrystallization. The ligand is produced by a high-efficiency one-pot process that complies with atom economy principles, with water as the only stoichiometric by-product. This sustainable approach reduces the E-factor and obviates the need for toxic auxiliary chemicals or intricate purification processes, aligning with recent developments in green Schiff base chemistry and sustainable synthetic design^55,56^.Conversely, several documented reagents in Table 5 need multi-step syntheses, stringent temperature regulation (e.g., diazotization for azo dyes like ATAP and ANPAP), hazardous substances (e.g., CS₂ in dithiocarbamates), or chromatographic purification, hence augmenting environmental impact and expense.

**Table 5:** Comparative data from recent studies on extraction and determination of Cu(II).

Sample matrix	Detection technique	Extraction step	Extractant	DL (µg L^− 1^)	RSD (%)	EF/PF	Refs.
Food, water and biological	UV-Vis	CPE	ATAP/Triton X-114	1.2	1.25	125	^28^
Environmental, water and biological	CPE	CPE	ANPAP/ Triton X-114	1.2	1.25	250	^30^
Water	FAAS	NADES- DLLME	NADES (menthol and decanoic acid)	1.9	< 2.3	9.8	^59^
	FAAS	DLLME	5-BrPADAP / CHCl_3_ and acetone.	1.4	< 6.5		^60^
Water, blood	FAAS	CP-DLLME	Cetyl pyridinium naphthenate	27	0.15	40	^61^
Water	FAAS	IL-DLLME	[C_4_mim][PF_6_]/DDTC	3.3	8.8	54	^62^
			[C_16_mim][Br]]/DDTC	5.1	10	27	
Lake water, seawater, tap water spiked	UV-Vis	IL-DLLME	[C_7_mim][PF_6_]/3-dimethylamino rhodamine/ Triton X-100	0.831	1.09	–	^63^
Spiked environmental water	FAAS	DLLM	1,5-Diphenylcarbazide	2	2.5	25	^64^
Food	FAAS	SPE	DCPIMI	1.9	2.1	225/35	^65^
Food	FAAS	SPE	BHAPDMPDI	1.8	< 5.0	34	^66^
Water	FAAS	EA-DES-LLME	DPC / DES (choline chloride and phenol)/ THF	2.9	1.30	78	^23^
Water and table salt	FAAS	VA-DLLME	PAN/Ethanol/1-Decanol	0.9	6.7	30	^68^
Water	UV-Vis	CPE	4-benzylpyridine dithiocarbamate/Triton X-100	1.4	2.1	10	^26^
Food and water	UV-Vis	RS-CPE	DDTC/ Triton X-100	0.4	3.7	18	^27^
Water and food	UV-Vis	IL-CPE	BHPIMP/Triton X-114/ [C_4_MIM][PF_6_]	0.6	≤ 2.0	20/60	Proposed method

Structurally, BHPIMP comprises azomethine (C =N) and phenolic donor groups that facilitate robust N, O-bidentate coordination with Cu(II), resulting in a stable chelate complex. The presence of a bromine substituent enhances the electron-withdrawing character, improving complex stability^57^, while also increasing ligand hydrophobicity^58^, which facilitates efficient phase transfer and extraction in cloud point systems.

Analytically, as shown in Table 5, the proposed BHPIMP-based IL–CPE method offers a low detection limit (0.6 µg L⁻¹) and excellent precision (RSD ≤ 2.0%), matching or outperforming more complex methods such as DLLME, SPE, and NADES-based systems. It also demonstrates high accuracy, good recovery, and a high preconcentration factor while minimizing the use of hazardous organic solvents^26–28,30,59–68^.

In addition, The proposed IL-CPE–spectrophotometric method demonstrates significant advantages in terms of operational simplicity and analytical throughput. The procedure involves minimal instrumentation, straightforward sample preparation, and relatively short analysis time, enabling the processing of multiple samples. Although spectrophotometry does not offer the multi-element capability of techniques such as ICP-OES or AAS, it provides a cost-effective, accessible, and portable alternative suitable for routine and on-site analysis. These features enhance the practical applicability (Blueness) of the method and contribute positively to its overall Whiteness by balancing analytical performance with economic and operational efficiency.

## Future perspectives

The existing IL–CPE method exhibits adequate sustainability; however, enhancements may be realized through the utilization of more environmentally friendly ILs, such as cholinium-based ionic liquids, which present reduced toxicity and superior biodegradability compared to PF₆⁻-based systems. Nonetheless, their increased hydrophilicity may influence phase separation and extraction efficacy, necessitating further tuning. In addition, while the present study is based on laboratory spectrophotometric analysis, the proposed system could in future be adapted for smartphone-assisted colorimetric detection to enable portable and cost-effective on-site applications.

## Conclusions

In this study, a sustainable and high-performance method (IL-CPE) was successfully designed and validated for the specific preconcentration of Cu(II) ions in real water and vegetables matrices before spectrophotoemtric analysis. The suggested strategy integrates operational simplicity with enhanced analytical sensitivity, offering a DL (0.60 µg L⁻¹) and a substantial PF of 60. Beyond its sensitivity, the approach is characterized by minimal solvent and reagent consumption, cost-effectiveness, and rapid execution, making it suitable for routine analytical applications. A notable advantage is its high tolerance toward coexisting ions, enabling reliable Cu(II) quantification in complex matrices without significant interference. The procedure’s precision was confirmed by repeatability and reproducibility values with RSD% below 3.0%, demonstrating excellent methodological stability. The application to real samples and CRMs further verified its analytical accuracy and practical reliability for Cu(II) detection in environmental matrics. From a sustainability perspective, the developed protocol exhibited a favorable ecological profile. The greenness assessment yielded AGREE score of 0.68, AGREEprep index of 0.64, a ComplexMoGAPI score of 86, and AGSA score (73.61). Practical applicability, evaluated using BAGI, yielded a blueness score of 67.5, while CACI score (70). The RGB algorithm indicated an overall whiteness value of 82%. The suggested approach has significant efficacy in diminishing the carbon footprint, as evidenced by the greatest cumulative CaFRI ranking of 80. These complementary metrics collectively confirm that the proposed IL-CPE method achieves a balanced integration of analytical efficacy, environmental sustainability, and economic viability. It is also important to highlight that the proposed IL–CPE method also exhibits strong potential for automation and scalability. The simplicity of the extraction procedure, low reagent consumption, and short analysis time make it amenable to integration into flow-based or sequential injection systems. Moreover, the method can be efficiently scaled for high-throughput analysis through parallel sample processing using conventional laboratory equipment, without compromising analytical performance. Overall, the developed method represents a reliable, sensitive, and sustainable analytical approach for the determination of trace Cu(II). It aligns well with modern green analytical chemistry principles and offers strong potential for routine applications as well as large-scale analytical implementations.

## Supplementary Information

Below is the link to the electronic supplementary material.

Supplementary Material 1

## Data Availability

All data generated or analyzed during this study are included in this published article [and its supplementary information files].

## Abbreviations

LOD: Limit of detection
EF/PF: Enrichment factor/preconcentration factor
RSD: Relative standard deviation
Ref: Reference
FAAS: Flame atomic absorption spectroscopy
UV–VIS: Ultraviolet–visible spectroscopy
DLLME: Dispersive liquid–liquid microextraction
NADES-DLLME: Natural deep eutectic solvent-dispersive liquid–liquid microextraction
IL-DLLME: Ionic liquid based dispersive liquid–liquid microextraction
VA-DLLME: Vortex-assisted dispersive liquid–liquid microextraction
CPE: Cloud point extraction
SPE: Solid phase extraction
EA-DES-LLME: Emulsification assisted-deep Eutectic Solvent-Liquid–Liquid Microextraction
THF: Tetrahydrofuran
5-Br-PADAP: 2-(5-Bromo-2-pyridylazo)-5-(diethylamino)phenol
C4MIM-PF6: 1-Butyl-3-methylimidazolium hexafluorophosphate
C_16_C_4_Im-Br: 1-Hexadecyl-3-butylimidazolium bromide
BHAPDMPDI: Bis(2-hydroxyacetophenone)-2,2-dimethyl-1,3-propanediimine
DCPIMI: 3-((2,6-Dichlorophenyl)(1 H-indol-3 yl)methyl)-1 H-indole
DDTC: Diethyldithiocarbamate
PAN: 1-(2-Pyridylazo)-2-naphthol
[HMIM][FAP]: 1-Hexyl-3-methylimidazolium tris(pentafluoroethyl)trifluorophosphate
NPAN: 1-(4-Nitrophenylazo)-2-naphthol
ATAP: 2-Amino-4-(m-tolylazo)pyridine-3-ol
ANPAP: Amino-4-(3-nitrophenylazo)pyridine-3-ol
RS-CPE: Rapidly synergistic cloud point extraction
