# Supplementary material for: Sustainable ionic liquid-assisted cloud point extraction for enrichment of trace copper(II) in water and food samples prior to spectrophotometric determination
Source: Sci Rep. 2026 Jun 10;16:18006. doi: 10.1038/s41598-026-56689-x (PMC13254298; doi:10.1038/s41598-026-56689-x)
Supplement: Supplementary file 1 — Supplementary Material 1 [file 41598_2026_56689_MOESM1_ESM.docx]

Supplementary materials

*Apparatus*

Absorption spectra were recorded using a Shimadzu UV-1601 double-beam UV/Visible spectrophotometer (Kyoto, Japan), equipped with 10 mm quartz cells, featuring a wavelength accuracy of ±0.2 nm and a 2.0 nm bandwidth. The pH measurements were performed with an AD1000 digital pH meter (Adwa Instruments, Szeged, Hungary). Phase separation was facilitated using a centrifuge (Isolab GmbH, Eschau, Germany), and temperature control was maintained using a thermostated water bath (Memmert WNB7-45, Schwabach, Germany). Infrared (FT-IR) spectra were recorded in the 4000–400 cm⁻¹ range using a Nicolet iS10 spectrometer (Thermo Fisher Scientific, Waltham, MA, USA) via KBr pellets. ^1^HNMR spectra were obtained in DMSO-*d^6^* on a JEOL (500 MHz) spectrometer (Tokyo, Japan) at the Chemistry Department, Faculty of Science, Mansoura University.


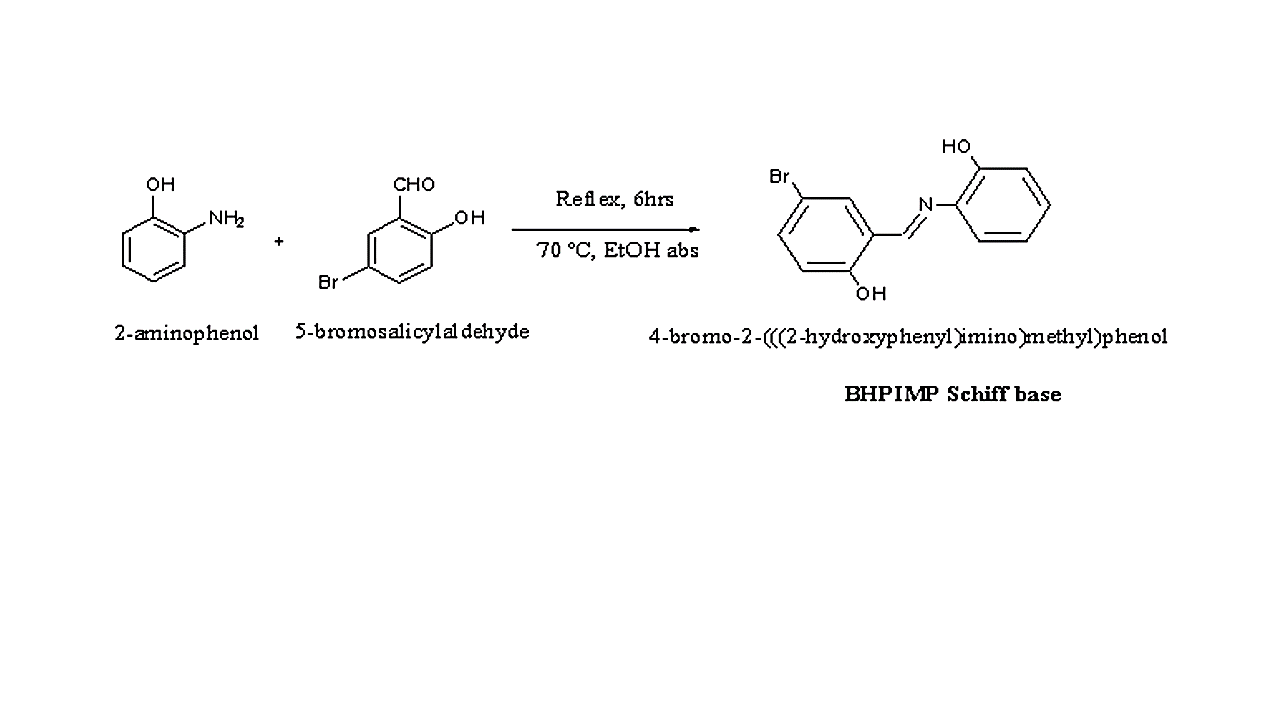


**Fig. S1.** General synthesis scheme for BHPIMP Schiff base.


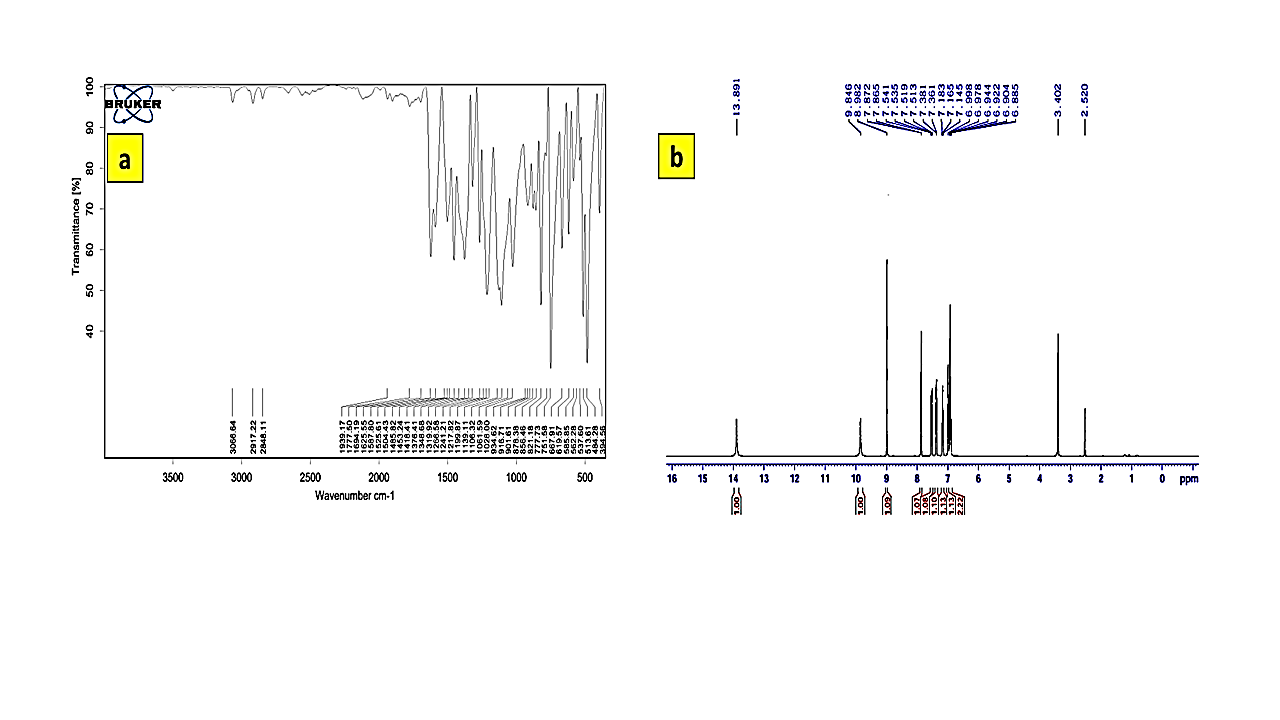


**Fig.S2**. (a) IR spectra and (b) ^1^H-NMR spectra of BHPIMP Schiff base.

| ****  **(a)** | ****  **(b)** |
| --- | --- |

**Fig.S3**. (a) Continuous variation (Job’s method) and (b) mole ratio method .
